# Youth Leisure-Time Sedentary Behavior Questionnaire (YLSBQ): Reliability and Validity in Colombian University Students

**DOI:** 10.3390/ijerph18157895

**Published:** 2021-07-26

**Authors:** Miguel Alejandro Atencio-Osorio, Hugo Alejandro Carrillo-Arango, María Correa-Rodríguez, Diego Rivera, José Castro-Piñero, Robinson Ramírez-Vélez

**Affiliations:** 1 Grupo de Investigación en Deporte de Rendimiento (GRINDER), Programa de Educación Física y Deporte, Universidad del Valle, Santiago de Cali 76001, Colombia; miguel.atencio@correounivalle.edu.co (M.A.A.-O.); hugo.carrillo@correounivalle.edu.co (H.A.C.-A.); 2 Grupo de Investigación en Actividad Física y Salud (GIAFS), Institución Universitaria Escuela Nacional del Deporte, Santiago de Cali 76001, Colombia; 3 Instituto de Investigación Biosanitaria Granada (IBIS Granada), Department of Nursing, Faculty of Health Sciences, University of Granada (UGR), 18001 Granada, Spain; macoro@ugr.es; 4 Department of Health Sciences, Public University of Navarre, 31000 Navarra, Spain; diegoriveraps@gmail.com; 5 GALENO Research Group, Department of Physical Education, Faculty of Education Sciences, University of Cádiz, 11510 Puerto Real, Spain; jose.castro@uca.es; 6 Instituto de Investigación e Innovación Biomédica de Cádiz (INiBICA), 11001 Cádiz, Spain; 7 Department of Health Sciences, Public University of Navarre, Navarrabiomed, CIBER of Frailty and Healthy Aging (CIBERFES), Instituto de Salud Carlos III, Pamplona, 31000 Navarra, Spain

**Keywords:** sedentary, screen time, reliability, validity, questionnaire, young adults, Latin American

## Abstract

Sedentary behavior (SB) is influenced by variations in social, cultural and economic contexts. This study assesses the test–retest reliability and validity of the Youth Leisure-time Sedentary Behavior Questionnaire (YLSBQ), a self-report tool that examines total and domain-specific SB in a cohort of young adults from Colombia. A cross-sectional validation study was conducted among 447 Colombian college students (52.8% men; mean (± standard deviation) age of 19.55 ± 2.54 years). To assess the reliability of the YLSBQ, Kappa statistics (k) were used. A confirmatory factor analysis (CFA) was conducted to determine validity. The Cronbach alpha for the 12 behaviors of the YLSBQ showed a good-to-excellent internal consistency (0.867, ranging from 0.715–0.935). Intraclass correlation coefficient (ICC) indicated that 10 items (83.0%) and two items (17.0%) showed excellent and good reliability, respectively. Furthermore, ICC between the total sedentary time was 0.926 (95% confidence interval [CI] = 0.912 − 0.939), which was interpreted as excellent. The goodness-of-fit tests provided evidence that overall, a four-factor solution was an adequate fit with the time scores. In conclusion, the YLSBQ could be considered a reliable, valid and usable tool for the assessment of SB in young adults in a Latin American country. We found that the psychometric properties of the questionnaire were similar to those of the original Spanish validation study.

## 1. Introduction

Sedentary behavior (SB) is defined on the basis of energy expenditure, i.e., any behavior in which energy expenditure is strictly below 2 METs (metabolic equivalent tasks) [1], or between 1 MET and 1.8 METs [2]. Nevertheless, since that criterion alone is not enough to clarify what SB is [3], the Sedentary Behavior Research Network (SBRN) undertook the Terminology Consensus Project and defined SB as “any waking behavior characterized by an energy expenditure ≤ 1.5 METs while in a sitting or reclining posture” [4]. Thus, common SBs include watching television (TV), using the computer, playing video games, using phones (collectively referred to as screen time), sitting in cars, and reading [5]. The definition of SB indicates that two behavioral components are crucial: an intensity/energy expenditure component and a postural component. 

Evidence supports that sedentary time has been linked to all-cause mortality, fatal and non-fatal cardiovascular disease, type 2 diabetes and metabolic syndrome [6,7]. Therefore, it has been of growing interest in recent years to examine SB, including how the behavior is assessed. To date, most epidemiological research regarding SB has been derived from studies using a single indicator for sedentary time, such as TV time or total sitting time. Hallal et al. [8] assessed the time spent sitting in 66 countries and found that 41.5% of adults worldwide spend four or more hours per day sitting. A national cross-sectional study using data from the National Health and Nutrition Examination survey (NHANES) showed that children and adults spent 54.9% of their monitored time, or 7.7 h/day, in SB [9].

Similarly, a study based on a large population (15–65 years) from Latin American countries reported that participants spent a total of 373.3 min/day engaged in total SB and the proportion of participants who reported >275 min of total SB per day was greater than half. It was notable that the highest values of domain-specific SB were reported for watching TV (146.3 min/day) [10]. In Colombia, data from the National Nutritional Survey (ENSIN 2015) showed that 56.9% of the participants between the ages of 18 to 64 years spend ≥2 h per day engaged in recreational screen time, such as a TV viewing, video game playing, computer use/tablets, or cell phones [11]. Please note that in this study, physical activity was assessed by Youth Risk Behavior Surveillance System (YRBSS) developed by the Centers for Disease Control and Prevention (CDC) that monitor six health-related categories but that it is not specific to assessing SB.

Considering that SBs are complex behaviors, their assessment remains a challenge. The methods used to measure SB include subjective approaches, such as diaries and self-report questionnaires, which have been mainly focused on TV viewing or other screen-based behaviors, and objective methods, including accelerometers, posture monitors, heart rate (HR) monitoring and combined sensing, multi-unit monitors [12]. However, these objective approaches are too time or resource intensive for inclusion in population-level health surveys and studies [12,13,14]. Therefore, examining the reliability and validity of self-report tools that provide information about mode and domains of SB questionnaires is of special interest.

The Youth Leisure-time Sedentary Behavior Questionnaire (YLSBQ) is a low-cost, simple, self-report tool for the assessment of several SBs. A previous Spanish study adapted and validated a YLSBQ version in a sample of 194 youth, aged 10–18 years [15]. This version of the YLSBQ reported moderate-to-substantial agreement for most (91%) of the items (*k* = 0.43 − 0.74; ICC = 0.41 − 0.79) and a moderate level of criterion validity (r = 0.36; *p* < 0.001), which was slightly higher than reported in some previous SB questionnaires for young people [16]. In recent years, Latin America has experienced positive changes in public transport systems, rapid urbanization and migration, increasing levels of ultra-processed food in the diet, with a parallel decrease in levels of physical activity, due to the mechanization of both daily work and leisure time. Moreover, the social, cultural and economic environments of Latin American countries differ from the European context [17]. 

Additionally, the examination of health lifestyles including SB in young adulthood, which is considered a transitional period in the domains of work, school, family and relationships, is of special interest. This period of life has important implications for patterns of health-related behavior and trajectories of adult health [18]. In this context, it is especially relevant to examine the reliability and validity of a SB tool in a specific cohort of young adults from Latin America. Thus, the aim of this study was to assess the test–retest reliability and validity of the YLSBQ in a cohort of young adults from Colombia.

## 2. Materials and Methods

### 2.1. Design and Sample

This cross-sectional validation study was conducted within the FRICAUN study (in Spanish, Factores de RIesgo CArdiovascular en UNivesitarios), which was a non-representative survey conducted in 2020 on collegiate students from Cali, Colombia. Detailed information about the objectives, design, and protocol has been published elsewhere [19]. Briefly, participants represented various academic disciplines on campus (e.g., engineering, physical education, natural sciences, health science, among others). All participants were socioeconomic status (SES) I–II (lower) to SES V–VI (higher, as determined by a scale of the Colombian government) in the capital of Cali, Valle del Cauca Department, in the Pacific region. Exclusion factors included a clinical diagnosis of cardiovascular disease, type 1 or 2 diabetes mellitus, pregnancy, the use of alcohol or drugs, and, in general, the presence of any disease directly associated with eating disorder (i.e., anorexia nervosa, bulimia, and others). A comprehensive verbal description of the nature and purpose of the study was given to the collegiate students and written informed consent was obtained from all participants. Participants were volunteers and did not receive any financial incentive for their involvement in the study.

For this study, we only used data from participants who reported 12 items of the questionnaire (*n* = 447; 52.8% men; mean age of 19.55 ± 2.54 years). Ethical approval for the whole project was obtained from the Ethics Committee of the Universidad del Valle (ID-001-020, Internal Code 233-019 of 18 March 2020). The study was conducted according to the ethical standards established in the 1961 Declaration of Helsinki (as revised in Hong Kong, China in 1989 and in Edinburgh, Scotland, in 2000). 

### 2.2. Variables and Measurements

A detailed description of variables and measurements has been published elsewhere [19]. Briefly, a trained staff interviewed participants individually, collected data on their SBs, and recorded anthropometric data measurements. Height was measured by a Seca stadiometer (Seca^®^ 274, Hamburg, Germany). Body weight (kg) was measured using an electric scale (Model Tanita BC-420^®^, Tokyo, Japan). Body mass index (BMI) was calculated using the formula proposed by Quetelet, in which BMI = body weight (kg)/height (m^2^). Participants were asked about their socioeconomical status. The testing procedure was conducted from October to November 2019.

This article describes the validation of the YLSBQ questionnaire designed by the Cabanas-Sánchez et al. [15] in the Spanish population aged 10–18 years. This questionnaire contains 12 items and was administered via the internet on two occasions one week apart. The participants were asked to think back over the previous week and report the estimated average time devoted to each behavior during weekdays and weekend days, separately. The answer options were none, 30 min, 1 h, 2 h, 3 h, 4 h and 5 h or more. The average time per day spent on each behavior and composite category was calculated as follows: [(weekday time × 5) + (weekend time × 2)]/7. A total sedentary time score was obtained by adding together the time reported for the 12 sedentary behaviors including screen time, non-screen sedentary time (NSST)-educational, NSST-social, and NSST-others. A detailed description of YLSBQ has been published elsewhere [14]. The instrument was submitted to a seven-day test–retest reliability study in a sample of 447 individuals.

### 2.3. Statical Analysis 

#### 2.3.1. Test–Retest Reliability

The weighted *k* coefficients with quadratic weights were computed for individual items. The internal consistency of the full scale and for individual items (i.e., weekdays and weekend values) were calculated through the Cronbach’s alpha (α) statistic [20] on two occasions one week apart. The Intraclass Correlation Coefficient (ICC) was calculated for items and composite categories. The weighted k, ICC and α values were characterized as follows: ‘poor’ (≤0.40), ‘moderate’ (0.41–0.60), ‘good’ (0.61–0.80) or ‘excellent’ (≥0.81) [21]. These analyses were processed with the program MedCalc Statistical Software version 15.8 (MedCalc Software bvba, Ostend, Belgium; www.medcalc.org (accessed on 4 November 2020); 2015).

#### 2.3.2. Validity

A confirmatory factor analysis (CFA) was conducted on the YLSBQ to determine whether the original version’s previously identified factor structure emerged in the same manner for Colombian population [22]. A CFA tests the fit of a hypothesized pattern of relationships among manifest (observed) variables and latent (hidden) variables. A CFA was conducted, assuming a priori model fit composed by 12 behaviors (time score) and four factors: (1) screen time (watching TV/videos, playing computer/video games, internet surfing); (2) Non-Screen Sedentary Time (NSST)-Educational (doing homework/study with computer, doing homework/study without computer, reading for fun), (3) NSST-Social (sitting and talking, listening to music, talking on the telephone), and 4) NSST-Others (sitting to rest, doing cognitive hobbies, traveling on motorized transport).

Several indices were used to test goodness of fit: ratio $X^{2}$/df, comparative fit index (CFI), incremental fit index (IFI), goodness-of-fit index (GFI), adjusted goodness-of-fit index (AGFI), Bentler relative noncentrality index (RNI), Bentler–Bonett nonnormed fit index (NFI), root mean square error of approximation (RMSEA), and standardized root mean square residual (SRMR). These analyses were performed using R program 3.6.3 for Linux-Ubuntu (R Development Core Team, 2020) [23]. The *lavaan* library [24] was used to conduct the CFA analyses.

## 3. Results

### 3.1. General Characteristics

The characteristics of the participants are summarized separately for males and females in Table 1. The average BMI was 21.80 (3.58) kg/m^2^ for males and 22.11 (3.22) kg/m^2^ for females. Significant differences were observed between males and females, with respect to body weight (*p* = 0.001) and height (*p* < 0.001). More than half of college students reported a low SES (61.9%). 

### 3.2. Test–Retest Reliability

The test–retest weighted Kappa, ICCs and Cronbach alpha coefficients for individual behaviors and category (weekday and weekend) are included in Table 2. The Cronbach alpha for the 12 behaviors of YLSBQ showed a good-to-excellent internal consistency (0.867, ranging from 0.715–0.935). The test–retest reliability study by ICC indicated that 10 items (83.0%) and two items (17.0%) showed excellent and good reliability, respectively. The total sedentary time mean score of the first and second survey were 153.15 (52.23) and 143.88 (51.85), respectively. Furthermore, ICC between the total sedentary time was 0.926 (95% CI = 0.912–0.939), which was interpreted as excellent.

### 3.3. Validity

A CFA was conducted using data from the 12 behavior time scores and the four-factor structure. Diagonally weighted least squares estimation was used, and indicators were modeled as continuous variables. The goodness-of-fit tests provided initial evidence that overall, the four-factor solution was an adequate fit with the time scores, because the ratio of the $X^{2}$/degrees of freedom was 2.05 (critical ratio cut-off of 2.0), RMSEA of 0.047 [90% CI = 0.03–0.06] (an RMSEA ≤ 0.08 indicates an adequate fit), GFI = 0.967, AGFI = 0.947 and SRMR = 0.047. Other evidence that generally indicated that the four-factor solution was a good fit with the data were relative noncentrality index (0.926), incremental fit index (0.928), and comparative fit index (0.926), which were all above 0.90, the traditional cut-off establishing adequate fit. Item loadings on their latent constructs were statistically significant (*p* < 0.001), suggesting that all items were a good index of their respective latent construct. The values of the estimated parameters between latent constructs and items are presented in Table 3.

## 4. Discussion

SB is associated with a greater risk for several major chronic disease outcomes and cardiovascular and all-cause mortality [25]. Since SB is recognized as a public health issue, there is a scientific interest to assess SB and their implications in epidemiological research [26]. This study examined the psychometric properties of the YLSBQ in a population of young adults from Colombia and demonstrated that the YLSBQ had good-to-excellent validity and reliability in this specific population.

SBs are influenced by variations in social, cultural and economic contexts [10,27,28]. This study validates for the first time the YLSBG, a self-report questionnaire that examines total and domain-specific SB, in a low-middle income country in Latin American context. To date, the majority of studies conducted in young adults from Latin American have assessed SB using a single indicator [29]. However, estimating only television (TV) viewing or total sitting time may not provide enough information to capture the general components of SB [4]. It has also been reported that the inclusion of a single question might imply that results may be overestimated [30]. Additionally, considering the important changes in the patterns of health behaviors that occur during the transition to adulthood which are usually linked to weight gain and reduced physical activity [31], the examination of SB in young adults is particularly relevant. Therefore, to examine the validity (the degree to which the questionnaire measures what it claims to measure) and reliability (the degree to which a questionnaire can produce consistent and reproducible results) in a specific population of Colombian youths is of special interest. The use of this reliable subjective measurement may facilitate the assessment of SB in epidemiological population-based studies conducted of similar contexts. Moreover, the identification of domain-specific SB in Latin American young adults might promote the planification of public health interventions and policies to avoid sedentariness as a lifestyle among this population.

The original validation study showed moderate-to-substantial test–retest correlations for most of the items and total sedentary time for an average day showed a good reliability value [15]. In our study, the Cronbach alpha for the 12 behaviors of YLSBQ showed a good-to-excellent internal consistency (0.867, ranging from 0.715–0.935) and the test–retest reliability study by ICC between the total sedentary time was 0.926 (95% CI = 0.912–0.939), which was interpreted as excellent. This level of internal consistency is similar to that reported by Cabanas-Sánchez et al. [15]. However, authors such as Nunnally [32] have suggested that the total explained variance must be significantly high (70%) for the number of factors to be sufficient.

Thus, these findings support that the YLSBQ is able to consistently collect information on leisure-time SB in young adults from Colombia. The original sample validation was composed of participants aged 8–18 years, while our sample consisted of young adults aged 18–25 years. Also, differences between both countries in sociodemographic factors, such as country per capita income or access to social media at home, which have been related to sedentary time [10], may affect the variability of the results. However, our data match the psychometric theory by Costello [21], showing that all items were a good index of their respective latent construct and establishing adequate fit with values above 0.90.

This study was subject to certain limitations. First, private and official universities were underrepresented in the sample. Nevertheless, the sample was heterogeneous, including participants of different SES. This is especially relevant since lower SES has previously been associated with greater SB [10]. Moreover, it should be noted that this study was carried out among students from different faculties, educational programs and disciplines. However, note that to validate this questionnaire among young adults in a university setting is important since it has been reported that sitting time can exceed 9 h/day [33]. Second, we did not include other instruments to calculate concurrent and discriminant validity. Finally, evaluation of the convergent reliability would be needed once any longitudinal follow-up is to be conducted. In any case, future studies may investigate this issue further. 

The heterogeneity of the population in the current study was a strength for validation, and suggests that YLSBG can be used in Latin American young adults with different characteristics. The YLSBQ, as a self-report questionnaire, has inherent limitations. However, since the assessment of SB by objective measurements may not be feasible to carry out in epidemiological studies with large study cohorts [12,13,14], the main strength of this study was to provide information regarding validity and reliability of this questionnaire in a population of youths from Colombia. Its validation in a specific population of Latin American young adults might facilitate its use by researchers and physicians in clinical context since is an easy-to-use tool. However, the limitations described in this work do not compromise the results achieved in the population studied.

## 5. Conclusions

Overall, our findings suggest that the YLSBQ could be considered a reliable, valid and usable tool for the assessment of SB in young adults within a Latin American country. We found that the psychometric properties of the questionnaire were like those of the original Spanish validation study. We expect that the assessment of the reliability and validity of the YLSBQ facilitate its use in both clinical practice and epidemiological research, since this instrument might provide valid data that allow the development and implementation of strategies for physical activity promotion.

## Figures and Tables

**Table 1:** General characteristics of the participants.

Characteristics	Males (*n* = 252)	Females (*n* = 225)	Full sample (*n* = 477)
Ages, (years)	19.55 (2.54)	18.95 (2.88)	19.27 (2.25)
Body weight, (kg)	66.24 (12.65)	57.83 (9.25) *	62.29 (11.93)
Height, (m)	1.74 (0.06)	1.61 (0.06) *	1.68 (0.08)
BMI, (kg/m^2^)	21.80 (3.58)	22.11 (3.22)	21.94 (3.42)
Socioeconomic status, *n* (%)			
1–2 (low)	61.9	69.2	65.4
3–4 (medium)	33.8	29.5	31.7
5–6 (high)	4.4	1.3	2.9

Data are reported as mean values (standard deviation, SD) or percentages. 2-h cutoff point was used due to evidence of harm to the health of young people who have SB above that value. * Significant differences by sex (*p* < 0.05).

**Table 2:** Test–retest reliability of Youth Leisure-time Sedentary Behavior Questionnaire (YLSBQ).

	Test (SD)	Retest (SD)	Difference (SD)	Kappa (95% CI) ^a^	ICC (95%CI) ^b^	Cronbach’s Alpha
1. Watching TV/videos/DVDs						
School day	2.75 (1.33)	2.57 (1.24)	0.18 (0.96) ^‡^	0.721 (0.680 to 0.761)	0.842 (0.811 to 0.868)	0.842
Weekend day	3.62 (1.61)	3.36 (1.48)	0.26 (1.01) ^‡^	0.782 (0.750 to 0.813)	0.884 (0.861 to 0.903)	0.884
2. Playing computer/video games (except Wii, Xbox Kinect or similar)						
School day	1.73 (1.22)	1.69 (1.19)	0.04 (0.68)	0.832 (0.788 to 0.876)	0.908 (0.890 to 0.923)	0.908
Weekend day	2.25 (1.66)	2.10 (1.55)	0.14 (0.78) ^‡^	0.876 (0.849 to 0.903)	0.935 (0.923 to 0.946)	0.935
3. Doing homework/study with computer						
School day	3.93 (1.51)	3.89 (1.35)	0.04 (1.02)	0.750 (0.712 to 0.787)	0.857 (0.829 to 0.880)	0.857
Weekend day	3.75 (1.57)	3.79 (1.47)	−0.04 (1.06)	0.756 (0.720 to 0.792)	0.860 (0.833 to 0.883)	
4. Internet surfing for fun (facebook, chat, tuenti, etc.)						
School day	4.05 (1.52)	3.90 (1.48)	0.14 (0.99) ^‡^	0.774 (0.738 to 0.810)	0.875 (0.849 to 0.896)	0.875
Weekend day	4.88 (1.57)	4.66 (1.52)	0.21 (1.01) ^‡^	0.777 (0.743 to 0.811)	0.879 (0.854 to 0.899)	0.879
5. Doing homework/study without computer						
School day	3.81 (1.36)	3.64 (1.25)	0.17 (1.00) ^†^	0.695 (0.649 to 0.742)	0.824 (0.789 to 0.854)	0.824
Weekend day	3.64 (1.44)	3.52 (1.38)	0.11 (1.08)	0.705 (0.663 to 0.748)	0.828 (0.794 to 0.857)	0.828
6. Sitting and talking with family or friends						
School day	3.26 (1.24)	3.13 (1.13)	0.13 (0.95) ^‡^	0.672 (0.611 to 0.733)	0.633 (0.558 to 0.695)	0.806
Weekend day	4.39 (1.46)	4.13 (1.39)	0.25 (1.01) ^†^	0.737 (0.697 to 0.776)	0.856 (0.826 to 0.880)	0.856
7. Sitting to rest (sunbathing, taking a nap, etc.)						
School day	2.89 (1.29)	2.80 (1.24)	0.08 (0.91) ^†^	0.741 (0.695 to 0.786)	0.852 (0.822 to 0.877)	0.852
Weekend day	3.79 (1.54)	3.60 (1.47)	0.18 (1.04) ^‡^	0.755 (0.718 to 0.792)	0.864 (0.836to 0.887)	0.864
8. Reading for fun						
School day	2.75 (1.30)	2.75 (1.20)	−0.00 (0.90)	0.741 (0.692 to 0.789)	0.851 (0.820 to 0.876)	0.851
Weekend day	2.99 (1.48)	2.88 (1.37)	0.10 (0.87) ^†^	0.811 (0.775 to 0.847)	0.897 (0.876 to 0.914)	0.897
9. Listening to music (without doing anything else)						
School day	2.37 (1.24)	2.39 (1.16)	−0.01 (0.88)	0.728 (0.674 to 0.782)	0.842 (0.810 to 0.869)	0.842
Weekend day	2.89 (1.45)	2.85 (1.41)	0.03 (0.97)	0.770 (0.729 to 0.812)	0.870 (0.844 to 0.892)	0.870
10. Talking on the telephone or send messages, WhatsApp, etc.						
School day	4.10 (1.72)	3.97 (1.58)	0.13 (0.95) ^†^	0.830 (0.803 to 0.856)	0.832 (0.801 to 0.858)	0.908
Weekend day	4.64 (1.79)	4.45 (1.71)	0.19 (0.98) ^‡^	0.838 (0.814 to 0.862)	0.914 (0.897 to 0.929)	0.914
11. Doing cognitive hobbies (doing puzzles, playing cards, doing crossword puzzles, etc.)						
School day	1.55 (0.82)	1.61 (0.80)	−0.06 (0.76)	0.555 (0.480 to 0.630)	0.715 (0.657 to 0.763)	0.715
Weekend day	1.81 (1.06)	1.87 (0.97)	−0.05 (0.85)	0.644 (0.582 to 0.706)	0.784 (0.740 to 0.820)	0.784
12. Traveling on motorized transport (car, bus, train, subway or motorbike)						
School day	3.54 (1.39)	3.65 (1.31)	−0.11 (0.90) ^†^	0.772 (0.733 to 0.811)	0.872 (0.847 to 0.894)	0.872
Weekend day	2.79 (1.34)	2.97 (1.38)	−0.17 (0.95) ^‡^	0.749 (0.706 to 0.792)	0.860 (0.832 to 0.884)	0.860

SD: standard deviation, ^a^ quadratic weights, ^b^ estimates the reliability of averages of *k* ratings. All *p* < 0.01. ^†^ Denotes statistical significance at the *p* < 0.05 level; ^‡^ denotes statistical significance at the *p* < 0.001 level.

**Table 3:** Parameter estimates to CFA of Youth Leisure-time Sedentary Behavior Questionnaire (YLSBQ).

Parameter Estimates	Estimate	Std Error	Pr (>|z|)
Watching TV/videos/DVDs: (Factor 1)	0.318	0.044	<0.001
Playing computer/video games (except Wii, Xbox Kinect or similar: (Factor 1)	0.009	0.038	0.818
Internet surfing for fun (facebook, chat, tuenti, etc.): (Factor 1)	0.668	0.091	<0.001
Doing homework/study with computer: (Factor 2)	0.543	0.077	<0.001
Doing homework/study without computer: (Factor 2)	0.672	0.094	<0.001
Reading for fun: (Factor 2)	0.441	0.067	<0.001
Sitting and talking with family or friends: (Factor 3)	0.486	0.050	<0.001
Listening to music (without doing anything else): (Factor 3)	0.329	0.042	<0.001
Talking on the telephone or send messages, WhatsApp, etc.: (Factor 3)	0.628	0.054	<0.001
Sitting to rest (sunbathing, taking a nap, etc.): (Factor 4)	0.424	0.098	<0.001
Doing cognitive hobbies (doing puzzles, playing cards, doing crossword puzzles, etc.): (Factor 4)	0.121	0.038	0.001
Traveling on motorized transport (car, bus, train, subway or motorbike): (Factor 4)	0.246	0.058	<0.001

## Data Availability

The data presented in this study are available on request from the corresponding author. The data are not publicly available due to ethical and private reasons.
